# High Connectivity in *Rastrelliger kanagurta*: Influence of Historical Signatures and Migratory Behaviour Inferred from mtDNA Cytochrome *b*

**DOI:** 10.1371/journal.pone.0119749

**Published:** 2015-03-18

**Authors:** Noor Adelyna Mohammed Akib, Bui Minh Tam, Preeda Phumee, Muchlisin Zainal Abidin, Saied Tamadoni, Peter B. Mather, Siti Azizah Mohd Nor

**Affiliations:** 1 Centre for Global Sustainability Studies, Universiti Sains Malaysia, Penang, Malaysia; 2 College of Aquaculture and Fisheries, Can Tho University, Can Tho city, Vietnam; 3 Faculty of Science and Fisheries and Technology, Rajamangala University of Technology Srivijaya Trang Campus, Trang, Thailand; 4 Department of Aquaculture, Faculty of Marine and Fishery, Universitas Syiah Kuala, Jl. T. Nyak Arief Darussalam, Banda Aceh, Indonesia; 5 Persian Gulf and Oman Sea Ecological Research Institute, Bandar Abbass, Iran; 6 Earth, Environmental and Biological Sciences, Queensland University of Technology, Brisbane, Queensland, Australia; 7 School of Biological Sciences, Universiti Sains Malaysia, Penang, Malaysia; 8 Centre for Marine & Coastal Studies, Universiti Sains Malaysia, Penang, Malaysia; Chinese Academy of Fishery Sciences, CHINA

## Abstract

Phylogeographic patterns and population structure of the pelagic Indian mackerel, *Rastrelliger kanagurta* were examined in 23 populations collected from the Indonesian-Malaysian Archipelago (IMA) and the West Indian Ocean (WIO). Despite the vast expanse of the IMA and neighbouring seas, no evidence for geographical structure was evident. An indication that *R*. *kanagurta* populations across this region are essentially panmictic. This study also revealed that historical isolation was insufficient for *R*. *kanagurta* to attain migration drift equilibrium. Two distinct subpopulations were detected between the WIO and the IMA (and adjacent populations); interpopulation genetic variation was high. A plausible explanation for the genetic differentiation observed between the IMA and WIO regions suggest historical isolation as a result of fluctuations in sea levels during the late Pleistocene. This occurrence resulted in the evolution of a phylogeographic break for this species to the north of the Andaman Sea.

## Introduction

In the marine realm, fish are abundant and ubiquitous and observed levels of genetic differentiation among populations are often low [1]. This pattern results from combined effects of large spawning population sizes that limit genetic drift effects, the apparent absence of physical barriers to dispersal for many species in their environment and presence of highly dispersive life history stages that contribute to high rates of gene flow among populations [2]. With no obvious major physical barriers to gene flow in the marine environment, genetic homogeneity or absence of spatial patterns in allele or haplotype distributions among geographical populations of marine species is expected and is often confirmed in genetics studies [3–4].

However, despite an apparent lack of any physical barriers to gene flow, many marine taxa have often been reported to show population structure [5–11]. Where structured populations are detected, this has been attributed to a number of factors including; historical and contemporary interplay among a complex set of ecological, demographic, behavioural, genetic, oceanographic, climatic and/or tectonic processes [12]. Evidence for population structure in several marine species has been reported in this region. A study of *Plectorhinchus flavomaculatus*, a coral reef fish sampled from the South China Sea in the Xisha, Zhongsha and Nansha archipelagos examined variation in the mtDNA control region and identified two discrete lineages [11]. A population pattern of ‘isolation by distance’ was observed in this species. The authors hypothesized that populations may have diverged in different glacial refuges during the Pleistocene at times of lower sea levels. Genetic relationships among northern populations of the six bar wrasse, *Thallasoma hardwicki* were examined from six localities in the South China Sea and three localities from the Solomon Islands in the South Pacific Ocean [13]. Three major groups were detected; a north-central group comprised of northwestern Taiwan and northern Vietnam; a southwestern group in southern Vietnam; and a southern group that included the central Philippines. Differentiations among these regions were attributed to limitations on gene flow that result from the impacts of sea surface currents. In addition, a study of the crimson snapper, *Lutjanus erythropterus* [8] reported that populations in East Asia were divided into two major clades: an eastern group that included populations in the western Pacific Ocean and the East Sea, and a South China Sea group that included populations from northern Malaysia extending to the South China Sea. The study suggested that limited gene flow between the eastern region and the South China Sea contributed to the observed geographical subdivision. Significant population structure was also evident in the white pomfret, *Pampus argenteus*, with three distinct clades (the South China Sea, the Arabian Sea and the Bay of Bengal) across the Indo-West Pacific region [14]. Late Pleistocene population expansions were hypothesized to have contributed to the reported population structure in this species.

The Indian mackerel, *Rastrelliger kanagurta* is an epipelagic Scombrid that occurs widely across the Indo-West Pacific from South Africa, the Seychelles and the Red Sea, east to Indonesia and northern Australia to Melanesia, Micronesia, Samoa, China and the Ryukyu Islands of southern Japan. It is also believed to have entered the eastern Mediterranean Sea via the Suez Canal [15]. It is one of three species in the genus *Rastrelliger* that also includes *R*. *brachysoma* and *R*. *faughni*. In the seas surrounding Malaysia, *R*. *kanagurta* is widespread across the northwest and east peninsular Malaysia, Sabah and Sarawak [16] where it is one of the most commercially important marine resources [17]. This species is a fast swimmer because of a fusiform and streamlined body and therefore highly migratory [18].

To date, there has only been limited study of the population genetics of *R*. *kanagurta*. These have included investigations of west peninsular populations employing RAPD markers [19] and mtDNA D-loop region [20]. A similar study, also based on RAPD markers examined three populations of *R*. *kanagurta* from the Indian Peninsular [21] while in another study allozyme markers (five enzymes and a single sarcoplasmic protein) were employed to investigate population structuring along the coastal waters of India and the Andaman Sea [22]. All studies [20–22] reported essentially panmixia among sampled populations, although weak population structure was detected between northern and southern populations of *R*. *kanagurta* in the Strait of Malacca off the coast of Perak [19].

Genetic markers including; allozymes, mtDNA and microsatellites have been employed widely as markers to investigate the population structures of many marine fish taxa [23–25]. Different types of markers however, can sometimes produce patterns of population structure that are not concordant [23]. In the current study, mtDNA cytochrome *b* (Cyt *b*) was utilised to assess population structure in *R*. *kanagurta*. This marker was chosen because Cyt *b* contains both slowly evolving codon positions, which are useful in phylogenetic studies and rapidly evolving codon positions that are useful for population studies [4, 26–27]. These characteristics make mtDNA Cyt *b* a suitable marker for studies of both phylogenetic and population genetic studies of fish [27–28].

The current study focused on examining genetic variation in *R*. *kanagurta* populations in waters that form part of a major marine biodiversity hotspot in the Indonesian—Malay Archipelago (IMA). These include the Strait of Malacca (an extension of the Indian Ocean), the South China Sea, the Sulu Sea and the Celebes Sea. In addition, four populations outlying this area were included as reference populations and to provide a wider coverage of the species natural distribution. These were from Can Tho, Vietnam, represented the northern South China Sea and two populations from Trang, Thailand and Banda Acheh, Indonesia represented the East Indian Ocean (Andaman Sea). The fourth population from Bandar Abbas, Iran represented the West Indian Ocean and served as the outgroup. Variation in the complete Cyt *b* gene was utilized to examine genetic structure in *R*. *kanagurta* across this vast expanse of waters.

## Materials and Methods

### Sampling and DNA sequencing

**Fig 1:**
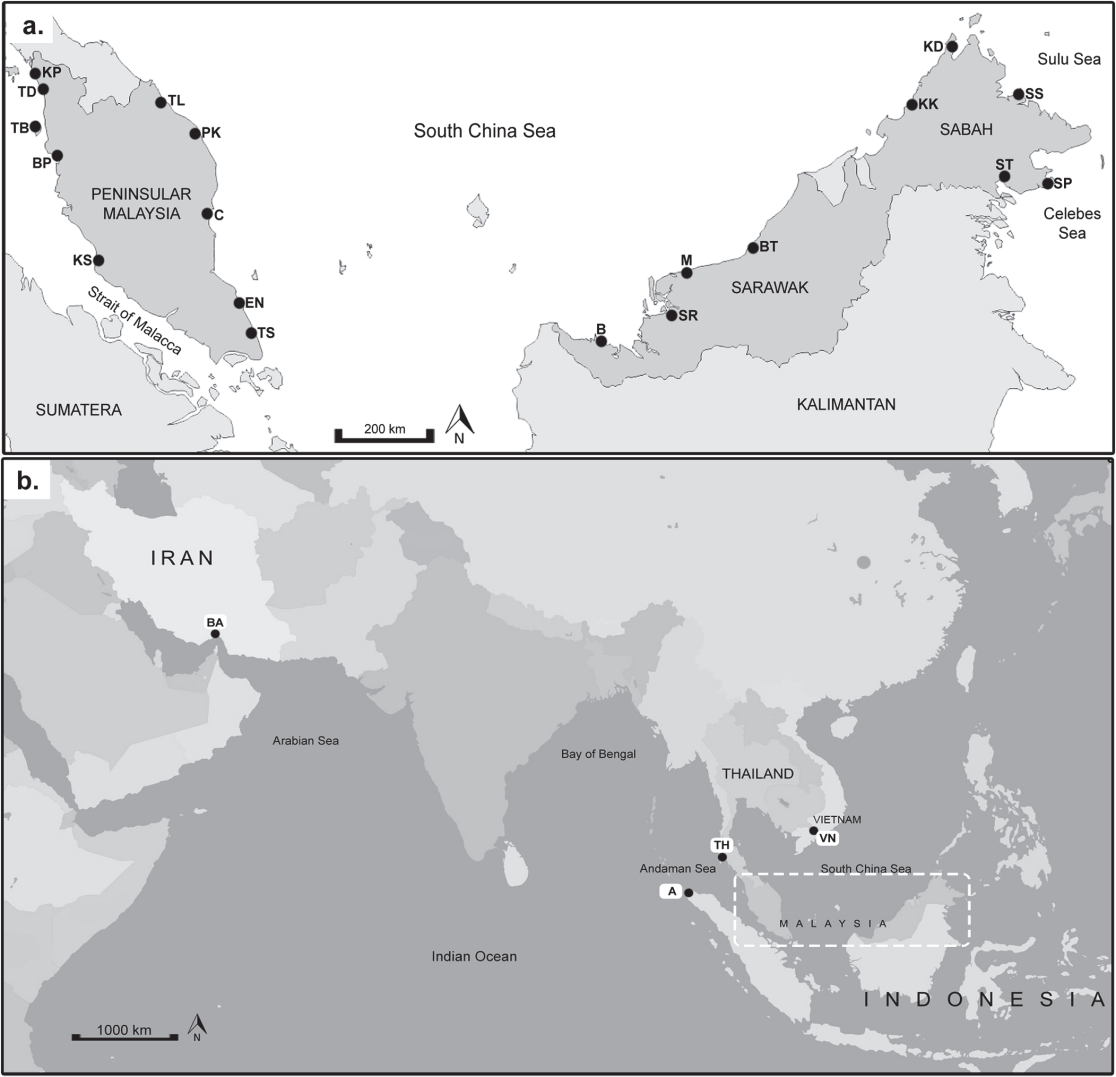
Sampling locations of *Rastrelliger kanagurta* (a) in the IMA subdivided into 5 regions; Region 1: Strait of Malacca (SOM)—Kuala Perlis (KP), Tanjung Dawai (TD), Bagan Panchor (BP), Teluk Bahang (TB) and Kuala Selangor (KS); Region 2: South China Sea 1 (SCS-1)—Tok Bali (TL), Kuantan (C), Endau (EN), Tanjung Sedili (TS); Region 3: South China Sea 2 (SCS-2)—Bintawa (B), Sarikei (SR), Mukah (M), Bintulu (BT), Kota Kinabalu (KK); Region 4: Sulu Sea (SLS)—Kudat (KD) and Sandakan (SS); Region 5: Celebes Sea (CS)—Semporna (SP) and Tawau (ST); (b) outside of the IMA subdivided into 3 regions; Region 3: South China Sea 2 (SCS-2, Table 1)—Can Tho, Vietnam (VN); Region 6: East Indian Ocean (Andaman Sea) (AS)—Trang, Thailand (TH); Banda Acheh, Indonesia (A); Region 7: West Indian Ocean (WIO)—Bandar Abbas, Iran (BA).

A total of 296 *R*. *kanagurta* individuals were collected from 19 locations within the IMA; Strait of Malacca (SOM), South China Sea (SCS), Sulu Sea and Celebes Sea between 2010 to 2011 (Table 1, Fig. 1A). In addition, three populations outlying this region, namely Banda Acheh, and Trang (both in the Andaman Sea) and Can Tho (South China Sea but outside of the IMA) were included. A West Indian Ocean (WIO), Bandar Abbas was also included. These additional populations to the IMA provided reference populations and a better geographical coverage, making in total, 342 individuals sampled from 23 locations (Fig. 1B). Populations were subdivided into seven geographical regions according to the seas for the analysis (refer Table 1). The South China Sea (SCS1 and SCS 2) was further subdivided due to its extensive wide geographical area. For the Malaysian samples, the samples were either obtained from fishing vessels operating in Zone A (from the shoreline to 5 nautical miles) or Zone B (from 5 to 12 nautical miles) at predetermined landing sites where catches do not overlap due to regulatory enforcement. Therefore, overlapping of populations from various landing sites could be disregarded. We get the fish samples throughout Malaysia with the help from Department of Fisheries through out Malaysia—Perlis, Kedah, Pulau Pinang, Perak, Selangor, Kelantan, Terengganu, Pahang, Johor, Sabah and Sarawak. For samples outside Malaysia, fin clips preserved in 99% ethanol were obtained through the assistance of local collaborators with permission from the respective authorities. All fishermen have the appropriate permits to fish. The field studies did not involve endangered or protected species. This study does not require an ethics statement because these samples are sea food product that can be found in the market.

**Table 1:** Sampling locations, coordinates, collection date and sample size of 22 *R*. *kanagurta* populations from the IMA and surrounding seas, and a single population from WIO.

Region	No	Population	Latitude (North)	Longitude (East)	Sampling date	N
Strait of Malacca (SOM)	1	Kuala Perlis (KP), Perlis	6°24’1”	100°7’51”	19.07.10	16
	2	Teluk Bahang (TB), Pulau Pinang	5°27’37”	100°12’25”	26.04.10	12
	3	Tanjung Dawai (TD), Kedah	5°40’48”	100°22’18”	27.01.11	19
	4	Bagan Panchor (BP), Perak	4°31’43”	100°38’2”	16.03.11	15
	5	Kuala Selangor (KS), Selangor	3°19’56”	101°15’5”	01.07.11	14
South China Sea (SCS-1)	6	Tok Bali (TL), Kelantan	5°53’24”	102°28’24”	27.05.10	15
	7	Pulau Kambing (PK), Terengganu	5°19’32”	103°7’53”	27.07.10	12
	8	Kuantan (C), Pahang	3°47’55”	103°19’24”	10.05.11	15
	9	Endau (EN), Johor	2°39’18”	103°37’23”	04.07.11	15
	10	Tanjung Sedili (TS), Johor	1°55’47”	104°6’48”	04.07.11	19
South China Sea 2 (SCS-2)	11	Bintawa (B), Sarawak	1°34’9”	110°22’33”	06.06.11	15
	12	Sarikei (SR), Sarawak	2°8’58”	111°31’9”	10.06.11	13
	13	Mukah (M), Sarawak	2°53’49”	112°5’44”	08.06.11	15
	14	Bintulu (BT), Sarawak	3°11’41”	113°5’23”	09.06.11	17
	15	Kota Kinabalu (KK), Sabah	5°58’58”	116°4’18”	09.07.10	16
	16	aCan Tho (VN), Vietnam	10°2’25”	105°47’0”	01.07.12	9
Sulu Sea (SLS)	17	Kudat (KD), Sabah	6°52’46”	116°50’45”	02.07.10	21
	18	Sandakan (SS), Sabah	5°50’20”	118°7’16”	03.07.10	15
Celebes Sea (CS)	19	Semporna (SP), Sabah	4°28’44”	118°37’4”	05.07.10	17
	20	Tawau (ST), Sabah	4°14’37”	117°53’4”	07.07.10	15
East Indian Ocean (Andaman Sea (AS)	21	Trang (TH), Thailand	8°15’18”	99°38’32”	01.10.12	7
	22	Banda Aceh (A), Indonesia	5°31’43”	95°24’6”	25.11.12	13
West Indian Ocean (WIO)	23	Bandar Abbas (BA), Iran	27°11’54”	56°16’3”	01.06.12	17
Total						342

^a^Can Tho was grouped in the SCS-2 region although it is not part of IMA.

In all sampling sites where they were physically collected, none of the samples were alive upon collection and they were not sacrificed for the purposes of this study. Fin clips were removed from the right pectoral fin from the dead samples and then preserved in 99% ethanol.

Extraction of DNA template was done using the Aqua Genomic DNA isolation kit (MultiTarget Pharmaceuticals, Salt Lake City, Utah 84116) as according to the manufacturer’s protocol. Isolated DNA template was PCR amplified for the complete mitochondrial Cyt *b*. Primer pair used for amplification was; L14317 (5’-CAG GAT TTT AAC CAG GAC TAA TGG CTT GAA-3’) and H15630 (5’-TTA ATT TAG AAT CCT AGC TTT GG-3’) (Takashima, unpublished). The PCR reaction mixture consisted of 2.5μL 10x PCR buffer (iNtRON), 3.3μL 25mM MgCl_2_ (iNtRON), 0.5μL 2.5mM of dNTP (iNtRON), 10 pM of each primer, 5U of *Taq* polymerase (iNtRON) and ∼50ng of genomic DNA. Amplification conditions were; initial denaturation at 94°C (2 min); 35 cycles of 94°C (20 sec), 50°C (20 sec), 72°C (1 min 10 sec) and a final extension at 72°C (5 min) before termination of the reaction at 4°C. Amplification of the 25 μL reaction volume was conducted in a G-Storm Thermal Cycler (MJ Research, Waltham, MA, USA). PCR products were purified using Promega Wizard SV Gel and PCR Clean-Up System (Promega Corporation, Madison, WI, USA) based on the manufacturer’s specifications. All purified products were sent for DNA sequencing (First BASE Laboratories Sdn. Bhd., Selangor, Malaysia and Centre for Chemical Biology, USM) in both the forward and reverse directions using Applied Biosystem ABI3730x1 capillary-based DNA sequencer.

### Data Analysis

Forward and reverse Cyt *b* sequences were edited in MEGA 5 [29]. Sequences were compiled and aligned using ClustalW that is integrated within MEGA 5 for unambiguous operational taxa units. Haplotype distributions for sampled populations were assessed using Collapse [30] and summarized using DnaSP 5.10 [31]. Phylogenetic relationships among haplotypes were constructed using Neighbour-Joining (NJ) method in MEGA 5 with a confidence level assessed using 1000 bootstrap replications. To view haplotype relationships, a phylogenetic network of all haplotypes was constructed based on median joining calculation in Minimum Spanning Network (MSN) [32]. The complete aligned dataset was then screened for nucleotide variable sites, parsimony informative sites, number of haplotypes, number of transitions and transversions and nucleotide frequencies in MEGA 5. The same software was used to estimate, genetic diversity within and among populations based on a K2P model. Genetic diversity, including gene diversity, nucleotide diversity and Theta S (θs) were retrieved in Arlequin 3.1 [33]. Historical demographic expansion was tested using Fu’s Fs [34] statistic in Arlequin 3.11 [33] in addition to Ramos-Onsins and Rosas’ R_2_ [35] in DnaSP 5.10 [31]. Significant values for R_2_ were calculated using coalescent simulations with 5000 replicate runs for each simulation. To detect whether sampled populations were demographically stable or expanding or decreasing over time, the demographic history of each sampled population was assessed applying Harpending’s raggedness index, H*ri* [36] implemented in Arlequin 3.1 [33] and mismatch distributions [37–39] implemented in DnaSP 5.10 [31]. Both Harpending’s raggedness index and mismatch distributions were used to detect whether the sequence data from each population deviated from what would be expected under a sudden population expansion model. A significant H*ri* value (P < 0.05) is evidence for rejecting this model [39]. Significance of the above tests was assessed using 10000 permutations. Past demographic events were also investigated based on the distributions of pairwise differences between sequences in Arlequin. Three parameters were estimated, θ_0_ and θ_1_(i.e. θ before and after the population growth) and τ (relative measure of time expressed in units of mutational time since the population expansion) [38, 40]. Values of τ were transformed to estimate real time expansion using the equation τ = 2μt, (μ = mutation rate of the sequence analysed, t = time since expansion). Changes in effective population size (Ne) across time were inferred using Bayesian skyline analyses. These analyses enable past demographic changes to be inferred from the current patterns of genetic diversity within a population [41]. BEAST v1.8 [42] was used to create the Bayesian skyline plots. To test for convergence, analysis was run for 10^8^ iterations with a burn-in of 10^7^ under the TN93 model, a strict molecular clock and a stepwise skyline model. Genealogies and model parameters were sampled every 1000 iterations. All operators were optimized automatically. Result of skyline plots were generated by Tracer1.5 [43].

Further analyses of genetic differentiation among populations were conducted for haplotype-based statistics (H_ST_) and sequence-based statistics (N_ST)_ [44] with significance levels assessed using permutation tests with 1000 replicates [45] in DnaSP 5.10 [31]. Estimates of gene flow (Nm) based on both haplotype and sequence statistics were derived employing the same program [45–46]. Genetic distance estimates between sampled populations were calculated using a Kimura 2 parameter distance method in MEGA 5. Analysis of molecular variance (AMOVA) was performed to estimate molecular variance among sampled populations using Arlequin 3.11 [33]. AMOVA partitions the total genetic variance into three measures of haplotypic diversity; *F*_ST_ describes variation between populations within total, *F*_SC_ describes variation among populations within region and F_CT_ describes variation among regions within total [33]. Spatial structure was examined further using Spatial Analysis of Molecular Variance (SAMOVA) v.1.0 [47] to identify groups of populations that were geographically homogeneous and maximally differentiated from each other and to identify genetic barriers between these groups [47].

## Results

All 342 individuals sampled from 23 localities amplified successfully for the Cyt *b* gene and a final sequence length of 1140 bp was obtained after alignment and editing of ambiguous sequences revealing 241 unique haplotypes. Sequences were deposited in Genbank with accession numbers JQ681542-JQ681735. A total of 220 variable sites were identified at the first and third base positions in the codon with a ratio of 1:8. Of these, 122 (10.7%) were parsimony informative. The ratio of transversion to transition substitutions of the entire data set was 1:11. Haplotype diversity was very high, ranging from 0.952 to 1.000, while nucleotide diversity was very low, ranging from 0.008 to 0.011 (Table 2).

**Table 2:** Parameters of variability estimates for *R*. *kanagurta* populations.

No	Pop	N	#V	H	Hd	K	π	Theta (S)
1	KP	16	44	15	0.992	11.733	0.010	13.260
2	TD	19	40	16	0.977	10.819	0.009	11.445
3	TB	12	35	12	1.000	11.242	0.010	11.590
4	BP	15	46	15	1.000	11.476	0.010	14.147
5	KS	14	45	14	1.000	12.462	0.011	14.150
6	TL	15	36	15	1.000	10.162	0.009	11.072
7	PK	12	35	11	0.985	10.379	0.009	11.590
8	C	15	41	14	0.991	10.429	0.009	12.609
9	EN	15	37	12	0.971	10.505	0.009	11.380
10	TS	19	41	18	0.994	10.058	0.009	11.731
11	B	15	36	13	0.981	10.019	0.009	11.072
12	SR	13	29	11	0.974	8.846	0.008	9.345
13	M	15	42	13	0.981	11.038	0.010	12.917
14	BT	17	45	17	1.000	11.103	0.010	13.310
15	KK	16	43	15	0.991	11.317	0.010	12.959
16	KD	21	52	18	0.981	10.867	0.010	14.454
17	SS	15	42	14	0.991	11.562	0.010	12.917
18	SP	19	42	15	0.965	9.363	0.008	12.017
19	ST	15	38	14	0.991	10.267	0.009	11.687
20	TH	7	30	6	0.952	13.190	0.011	12.245
21	A	13	38	13	1.000	10.436	0.009	12.245
22	VN	9	32	8	0.972	11.194	0.010	11.774
23	BA	17	51	15	0.978	10.772	0.009	15.086
Total Collection		322	238	236	^a^0.992	^a^11.559	^a^0.010	^a^12.391

Abbreviations: Sample size (N); Number of variable sites (#V); Number of haplotypes (H); Haplotype diversity (Hd); Mean number of pairwise differences (K); Nucleotide diversity (π); Expected heterozygosity per site based on number of segregating sites (theta, S)

^a^mean

Due to the higher number of unique haplotypes detected, only haplotypes shared among sites are shown for clarity (S1 Table). These unique haplotypes consist of mainly singletons (213/241–88.38%) and they are population specific (221/241–90.46%). Out of a total of 241 unique haplotypes, only 20 (9.54%) were found in more than a single region, with the rest being regionally specific. The BA (WIO) population from the Indian Ocean consisted mainly of singletons (16 of 17), all of which were private haplotypes to BA. As observed, no obvious population structure was detected, either related to the sea or with geographical distance. Several haplotypes were shared only among the IMA populations, but this was probably because of the low sample number of sites outside of the IMA waters as many other haplotypes were common among different seas (e.g. haplotypes 12, 52 etc.).

**Fig 2:**
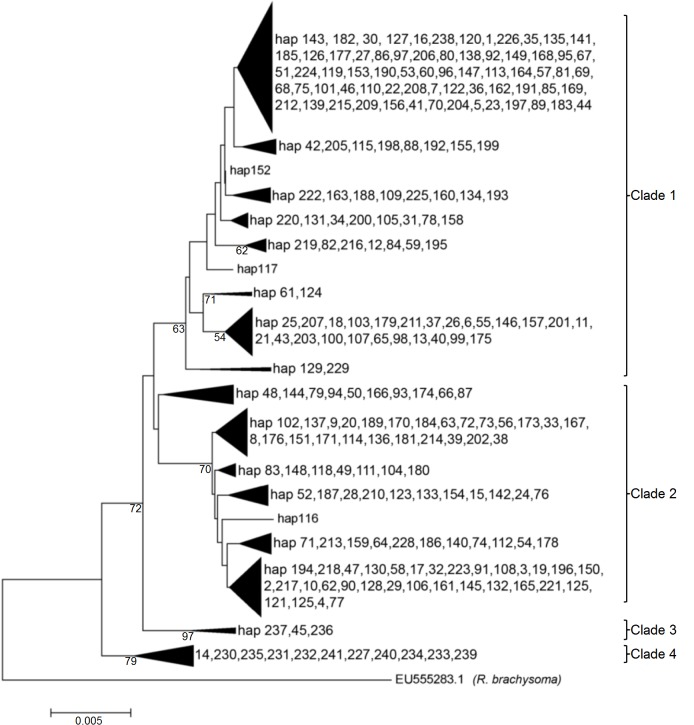
Evolutionary history as inferred using the Neighbor-Joining method (K2P distance) with 1000 bootstrap replicates.

**Fig 3:**
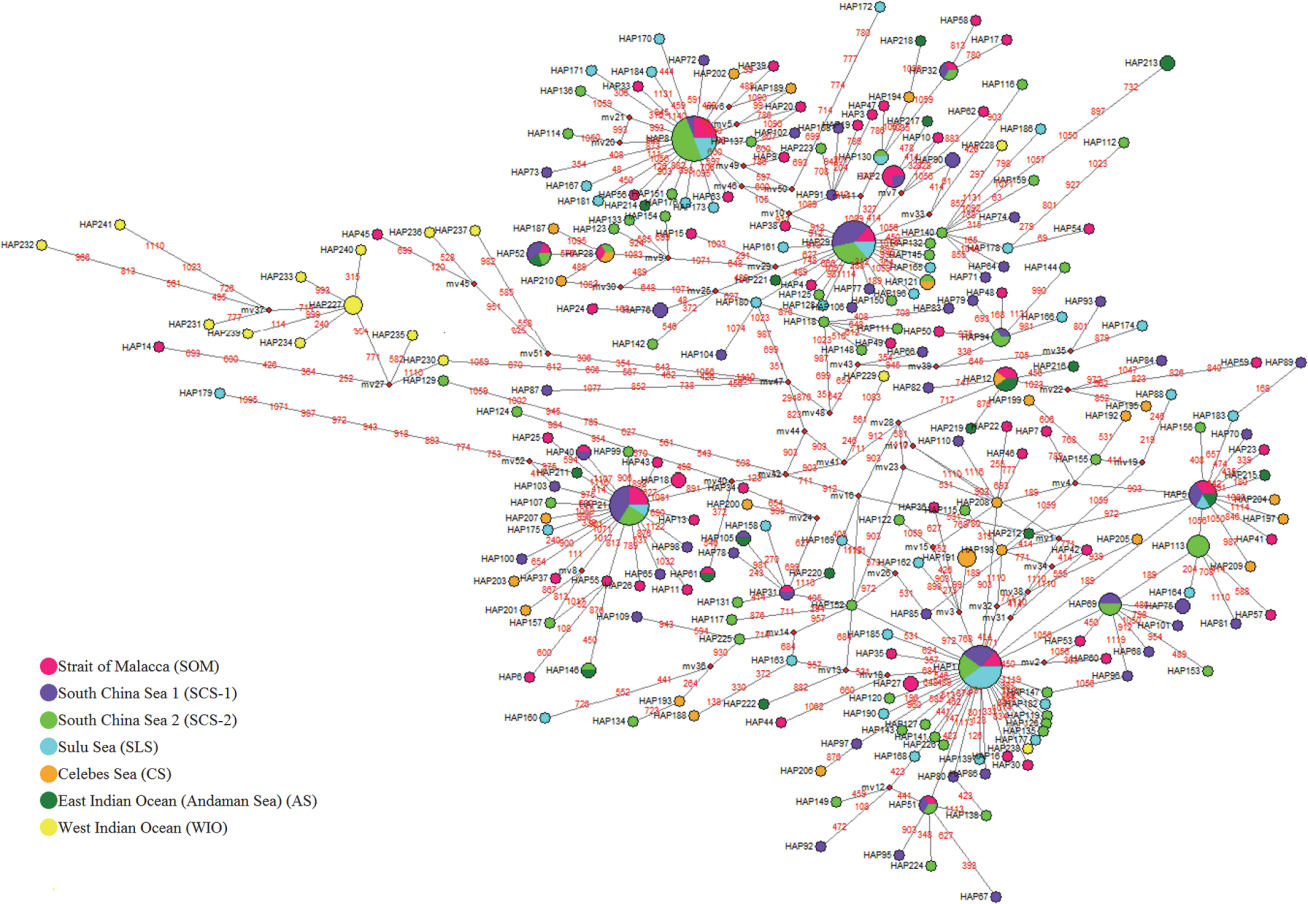
Minimum spanning network (MSN) inferred from complete mtDNA Cyt *b*. **Coloured close circles represent different regions (refer to legend).** mv = median vectors. The red circle in the figure represents the BA tip haplotypes.

The NJ tree was moderately supported with four lineages identified (Fig. 2). While no obvious geographical structure was evident for Clade 1 and 2, in contrast, Clades 3 and 4 were mostly composed of the Iranian population (western Indian Ocean) with only two representatives from the Strait of Malacca (haplotypes 14, 45 respectively). The minimum spanning network (MSN) produced a complex reticulation of 241 haplotypes (Fig. 3) with four major haplotypes. Two of the four highest frequency haplotypes were mainly formed of the SCS-2 sites (haplotypes 8 and 29). The other two were mostly represented by the SCS-1 populations (haplotype 21) and the SLS population (haplotype 1). According to the coalescent theory [48], common haplotypes internal to the network can be inferred to be ancestral, while tip haplotypes are derived or descendant from ancestral (internal) haplotypes. The Bandar Abbas (WIO) haplotypes are mostly at the tip. The occurrence of star-like patterns radiating from these major haplotypes suggest that *R*. *kanagurta* populations have undergone significant population size expansions in the relatively recent past [37,49].

Overall, 14 out of 23 population tested were significant with negative values for Fu’s Fs. This suggests historical population expansions at these sites [34] (Table 3) associated with an excess of recent mutations or rare alleles [34]. The Harpending raggedness index (H*ri* = 0.031, p>0.05) corroborated results from Fu’s Fs analysis indicating that some populations had expanded recently.

**Table 3:** Statistical tests of neutrality (Fu’s Fs) and demographic parameter estimates for *R*. *kanagurta*.

No.	Population	Tau	*Hri* index	Fu’s Fs	θ_0_	θ_1_	R_2_
1	KP	16.313	0.017	-4.997*	0.004	47.031	0.110
2	TD	15.430	0.022	-4.219	0.000	26.746	0.111
3	TB	15.023	0.030	-4.503*	0.004	30.371	0.128
4	BP	14.117	0.045	-6.710*	0.002	96.719	0.092
5	KS	15.711	0.034	-5.543*	0.007	42.937	0.106
6	TL	15.375	0.025	-7.355*	0.002	21.696	0.116
7	PK	15.600	0.042	-2.748	0.000	57.979	0.107
8	C	13.566	0.013	-4.802*	0.334	25.820	0.092
9	EN	13.830	0.023	-1.834	0.002	34.377	0.118
10	TS	13.336	0.013	-8.401*	0.000	30.527	0.092
11	B	15.059	0.025	-3.280	0.000	24.166	0.110
12	SR	0.607	0.026	-2.410	11.333	99999.000	0.124
13	M	15.434	0.043	-2.917	0.000	30.079	0.105
14	BT	16.342	0.015	-8.629*	0.000	37.477	0.094
15	KK	15.732	0.016	-5.710*	0.000	29.941	0.106
16	KD	16.113	0.010	-5.670*	0.002	26.795	0.079
17	SS	16.637	0.020	-4.350*	0.005	25.251	0.115
18	SP	4.104	0.037	-3.583	6.572	89.023	0.009
19	ST	14.736	0.011	-4.874*	0.000	23.730	0.107
20	TH	18.781	0.0930	0.627	0.005	40.437	0.157
21	A	3.240	0.036	-5.547*	10.593	99999.000	0.104
22	VN	15.762	0.086	-0.809	0.000	37.510	0.126
23	BA	0.854	0.021	-4.347	12.734	99999.000	0.0780
TOTAL		7.816	^a^0.031(0.662)	^a^-4.437*	^a^1.809	^a^13077.201	0.024(0.0002)*

Significant value p<0.05 (*) after FDR procedure α = 0.05.

^a^Mean

**Fig 4:**
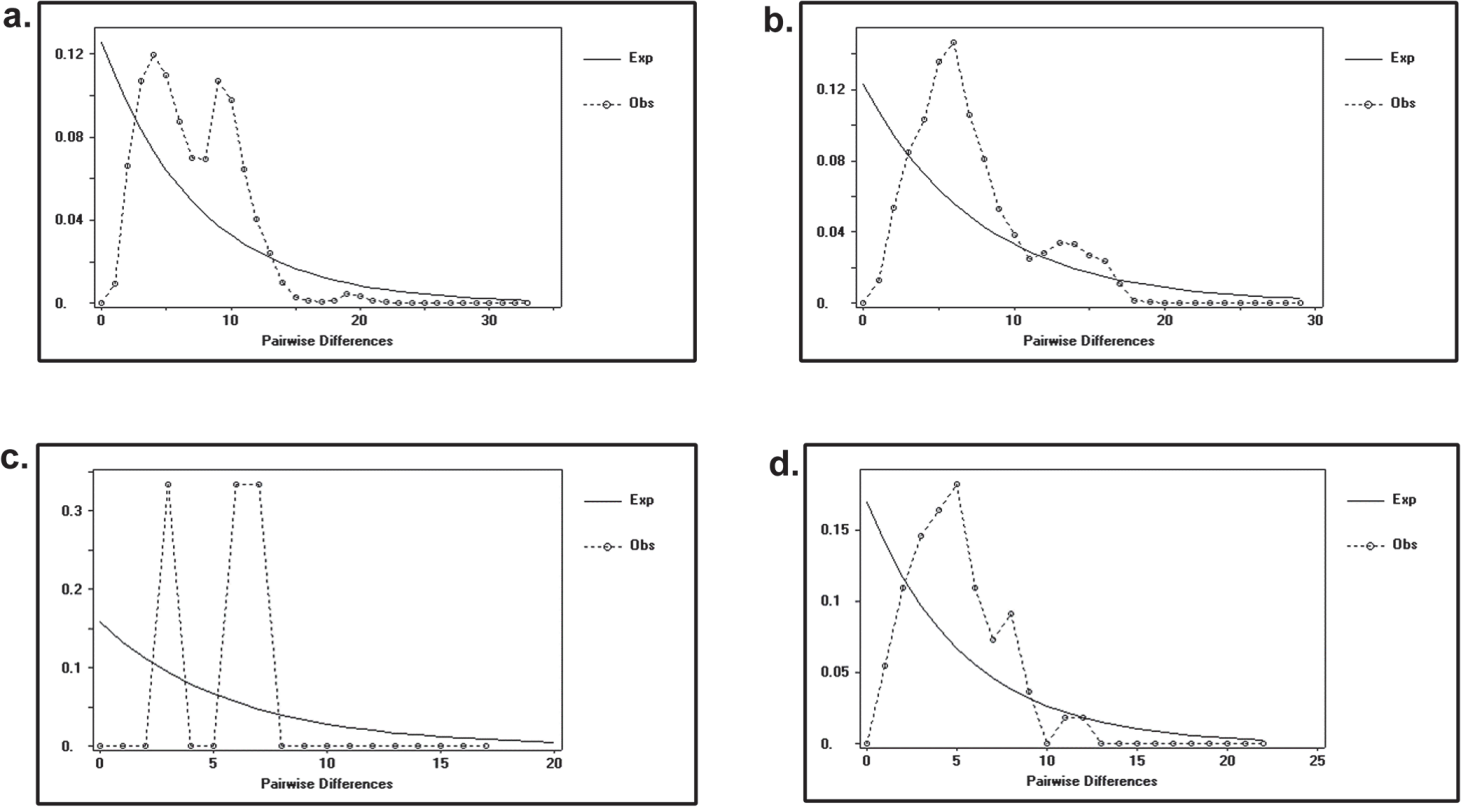
Mismatch distribution (pairwise number of differences) for the mtDNA Cyt *b* gene of *R*. *kanagurta* showing the expected and observed pairwise differences between the sequences with the respective frequency for (a) Clade 1(b) Clade 2 (c) Clade 3 (d) Clade 4.

**Fig 5:**
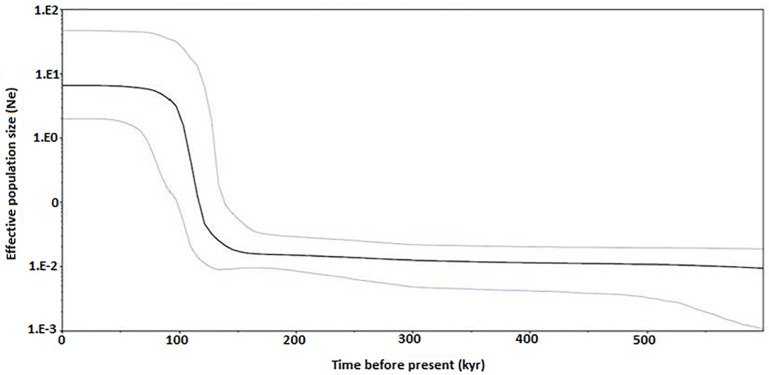
Bayesian skyline plot showing changes of effective population size (N_e_) through time inferred with Beast based on mtDNA Cyt *b* sequences. Black line represents median estimate N_e;_ light lines are the upper and lower 95% highest posterior density (HPD) limits of N_e._

Past population demographic of *R*. *kanagurta* matched the bimodal mismatch distribution for all four clades. Comparison between observed frequencies of pairwise differences with those expected under various demographic models [50] (Fig. 4) added support for a spatial expansion followed by a recent demographic expansion [50–51]. Bayesian skyline plot revealed an episode of abrupt demographic expansion around 100 to 120 thousand years (kyr) before present, which also falls into the Pleistocene period (Fig. 5). The flat skyline plot also indicates that the population has remained at a constant size over time before the expansion. Time since the population expansion was estimated to be 7.816/2u generations. Given a mutation rate of perciform Cyt *b* of 1–2% per million years [52–53], the *R*. *kanagurta* population expansion was estimated to have taken place 171,403 to 342,807 years bp.

AMOVA analysis performed on the 23 populations, subdivided into seven geographical groups according to the seas (group 1: SOM; group 2: SCS-1; group 3:SCS-2; group 4:SS; group 5: CS; group 6: AS; group 7: WIO). The South China Sea (SCS1 and SCS 2) was further subdivided due to its extensive wide geographical area. AMOVA analysis showed that among group variation was less than 1% (*F*_CT_ = 0.0023, p<0.05) but significant genetic differentiation was present between at least two groups. Genetic differentiation among populations within groups was 0.41% and significant (*F*_SC_ = 0.0041, p>0.05). Presumably this results from the divergence of the BA population from all other sampled sites when site BA was included in the analysis. Genetic variation within populations was high at 99.36% revealing that most of the variation was present within populations, while *F*_ST_ were low and not significant (*F*_ST_ = 0.0064, p<0.05). The analysis was repeated with the Iranian (BA) population (WIO) excluded to test. The test is to discover whether genetic differentiation was evident at finer spatial scales across the IMA and the surrounding seas (Trang, Banda Aceh—Andaman Sea of east Indian Ocean; Can Tho- South China Sea). The samples were divided into 6 groups; group 1: SOM; group 2: SCS-1; group 3: SCS-2; group 4: SS; group 5: CS, group 6:AS. The *F*_CT_ value for this additional analysis was not significant (p>0.05).

**Fig 6:**
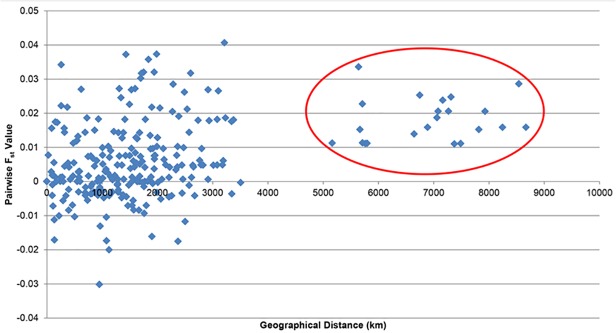
Scatter plot of geographical distance vs pairwise *F*_ST_ value of *R*. *kanagurta* individuals from Mantel test. The red circle indicates the western Indian Ocean population (Bandar Abbas, Iran).

Results of a Mantel test [54] supported previous outcomes showing no significant correlation between genetic differentiation (pairwise *F*_ST_ value) and geographical distance (r = 0.370, p>0.05, 1000 permutations) among the sampled populations (Fig. 6). The graph, however, did show two distinct clusters. The first cluster comprised all samples from the Strait of Malacca, South China Sea, Sulu Sea and Celebes Sea and the Andaman Sea (eastern Indian Ocean- Banda Aceh and Trang), while the second contained the western Indian Ocean population of Bandar Abbas. The Cyt *b F*_ST_ values were close to 0 (-0.030–0.042) (data not shown) and were not significant (p>0.05) after FDR correction (α = 0.05). An indication of minimal or no genetic structure present. Further analyses of genetic differentiations estimate based on H_ST_ (0.0024), N_ST_ (0.1581) and K_ST_* (0.0246) were very low and all were not significant, which also inferred a high level of gene flow among sampled *R*. *kanagurta* populations (Nm = 136.45 for haplotype-based statistic and Nm = 7.39 for sequence-based statistic).

Based on estimates of k = 2 to 8 in the SAMOVA analysis each group represented a statistical discrete phylogeographic grouping. However, k = 2 yielded the highest *F*_CT_ value (*F*_CT_ = 3.0374, p<0.05), a clear signal that there were two genetically different *R*. *kanagurta* stocks among the sampled populations, namely the IMA and adjacent regions and a western Indian Ocean population at Bandar Abbas.

## Discussion

In this study, high haplotype diversity, Hd values (ranging from 0.952 to 1.000) coupled with very low nucleotide diversity, π (ranging from 0.008 to 0.011) observed in most populations concord with reports from earlier studies of several pelagic marine fishes. Previous studies include the spotted mackerel, *Scomber australasicus* (Hd = 0.996, π = 0.007) [55]; wahoo, *Acanthocybium solandri* (Hd = 0.918, π = 0.006) [56]; yellow fin tuna, *Thunnus albacares* (Hd = 0.997, π = 0.035) and skipjack tuna, *Katsuwonus pelamis* (Hd = 0.999, π = 0.084) [57]. Population histories of species can be interpreted into four categories based on combinations of their Hd and π values from small to large [58]. In the case of *R*. *kanagurta*, high Hd and low π values suggest a recent population expansion from a low effective population size, i.e., rapid population growth after a bottleneck event with associated accumulation of many new mutations [59].

Four phylogenetic groups were evident in the Cyt *b* data for *R*. *kanagurta*. There were, however, no distinct geographical lineages present among the IMA and neighbouring marine populations in this study. All of the IMA sites are grouped in Clades 1 and 2 with no obvious pattern of geographic structuring. Clade 3 and 4 were almost exclusively composed of individuals from the western Indian Ocean at Bandar Abbas. Several Iranian individuals were also members of Clade 1 (hap 229, 238) and Clade 2 (hap 228). Two haplotypes present in the Strait of Malacca were members of Clade 3 (hap 45) and Clade 4 (hap 14), respectively. These indicate only very limited genetic exchange between the two regions. The divergence of these clades, however, may have happened in the past during the lowering of sea level during the Pleistocene period. Past geological and climatic events have undoubtedly played a major role in this inferred population expansion. The paleogeography of the IMA changed dramatically across the Quaternary period [60]. This area encompasses mainland Southeast Asia and Australasia, and is also known as the Indo-Australian Archipelago or East Indies. During the Pleistocene glacial periods, lowering of ocean levels periodically exposed the Sunda Shelf and Sahul Shelf within this archipelago. The former is an extension of the coastal shelf of Southeast Asia, including the Malay-Peninsula, Sumatra, Java and Borneo to Palawan. Fluctuations in sea levels during the Pleistocene epoch would have resulted in a significant impact on the dispersal of marine and terrestrial species. Thus shaping the modern genetic structures of many species that inhabit this region as we see it today [61–64]. This scenario had been hypothesized for many marine species including the redlip mullet, *Chelon haematocheilus* [65] and Japanese sea bass, *Lateolabrax japonicas* [66].

During the last Interglacial Period, there were marine connections between the Indian Ocean and the South China Sea via the Straits of Singapore [64]. These connections would have permitted extensive population expansion by *R*. *kanagurta* where previously they would have been isolated. Estimation of the time since expansion of *R*. *kanagurta* suggests that the timing of this event ranged from 171,403 to 342,807 years bp. The timing of the event is consistent with the occurrence of sea level rises cyclically during the late Pleistocene between 1,600,000 to 10,000 years ago [8,67]. Land bridges that were exposed during periodic declines in sea level prior to the last Interglacial period were likely to have hampered dispersal of *R*. *kanagurta* and led to smaller isolated populations. During that time, no barriers to dispersal by sea were present, at least between Peninsular Malaysia and the islands of the Riau Archipelago and Sumatra. Thus consequently permitting free migration of *R*. *kanagurta* populations across the region. The last glacial maximum (LGM), 30,000 to 19,000 years ago led to the final decline in sea levels to modern times [67–68]. A substantial area of the Sunda and Sahul shelves were exposed periodically as sea levels associated with glaciations declined up to 200m below their present level [69]. This process would also have hindered dispersal by many marine taxa. At this time, the adjacent South China Sea was significantly reduced in size and formed a semi-closed marginal sea and this exposed a large low gradient on the Sundaland craton[67]. During this time, the modern Malayan Peninsula, Borneo and Sumatra formed highlands to the South [70] producing a potential barrier to population migration.

Isolated marine populations at this time would have been subjected to increased levels of inbreeding. Potentially leading to declines in population levels of genetic variation and genetic structuring of populations during this short period of the LGM. There are also possibilities of individuals that may have dispersed to the east or west of the adjacent flooded basins. This pattern is not concordant however, with the pattern observed in *R*. *kanagurta*. They experienced almost complete panmixia evident in modern populations in SE Asia over a vast expanse of two oceans. It can be conjectured therefore, that the brief duration of LGM, followed by a rise of sea level approximately 18000 years ago until the present modern sea levels were reached, was insufficient for *R*. *kanagurta* to attain migration drift equilibrium. This would be manifested in low genetic differentiation and low genetic structuring among extant populations until the present time.

Similar scenarios have been hypothesized for many marine species present across this region. Examples include the Indo-Pacific butterfly fishes [71], pelagic scads, *Decapterus macrosoma* and *D*. *macarellus* [72] and Eastern little tuna, *Euthynnus affinis* [73]. Based on the results of the MSN analysis (Fig. 3) it is postulated that this expansion probably occurred from east to west as evidenced by the Sundaland origin of the four major haplotypes (ancestral) while all unique WIO haplotypes were tip haplotypes (descendant). Detailed examination of the MSN analysis revealed that two of the four putative ancestral haplotypes in the SCS-1 and SCS-2, based on the highest frequency suggest that the centre of origin for modern *R*. *kanagurta* populations were in the South China Sea from which populations could have radiated both eastwards and westwards.

Marine fishes with high dispersal potential typically display low levels of population genetic structure and associated high levels of ongoing gene flow [74]. This would produce shallow evolutionary trajectories and, potentially, limit or reduce adaptive divergence among local populations [7]. The overall low and non-significant *F*_*ST*_ values reported here among the 23 *R*. *kanagurta* populations are concordant with a pattern of Cyt *b* homogeneity and high gene flow (Nm = 136.45). The level of genetic differentiation among populations has a predictable relationship with rates of important evolutionary processes (migration, mutation, drift) [75]. Thus, a highly migratory species with large populations should show limited population differentiation. This is in contrast to species with small populations and reduced migration rates, where we might expect greater differentiation [75].

Several other investigations have also reported relative panmixia in marine species across this region. For example, an apparent lack of genetic structure in marine species present in the Coral Triangle region of Southeast Asia has also been reported in several marine taxa including; pelagic scads, *Decapterus macrosom*a and *D*. *macarellus* [72] and the eastern little tuna, *Euthynnus affinis* [73]. Slight population differentiation was observed between the IMA (and adjacent populations) with the WIO Bandar Abbas population. Moderate to high rates of gene flow among populations can prevent sub-population isolation, hence maintaining genetic variation levels and reducing inbreeding [76–77]. This, however, not always a simple relationship. The hierarchical AMOVA results here showed low but significant *F*_ST_ and *F*_CT_ outcomes. An indication of shallow genetic structure among populations of *R*. *kanagurta*. This was confirmed by spatial genetic heterogeneity (k = 2 in SAMOVA) between groups comprising populations in the IMA and surrounding seas *vs* a second group in the western Indian Ocean (Bandar Abbas, Iran). The Mantel test results did not support an ‘isolation by distance’ model. Thus a more plausible explanation for population differentiation between the two distinct groups of *R*. *kanagurta* was a result compounded by co-effects of various factors, including historical physical isolation during the Pleistocene epoch, larval dispersal factors and ocean currents.

Historical isolation would suggest the genetic differentiation evident between the IMA and WIO regions may have resulted from fluctuations in sea levels during the late Pleistocene. This led to a phylogeographic break for this species to the north of the Andaman Sea. Studies of other marine taxa across this region have reported co-effects on population differentiation of various factors. This was observed in the orange-spotted grouper, *Epinephelus coiodes* from China (South China Sea) and Malaysia through Indonesia (Southeast Asia) [78].

Ocean surface currents can also play an important role in marine larval dispersal. The surface current of the Indian Ocean is mainly under the influence of monsoon currents, also referred to as monsoon drift. It flows between the Bay of Bengal and the Arabian Sea [79]. In contrast, the seas surrounding Malaysia are affected by the Kuroshio current, the major surface current in the South China Sea [14]. These two sea surface currents could potentially contribute to population differentiation of eastern and western populations of *R*. *kanagurta*.

The Cyt *b* analysis of *R*. *Kanagurta* populations in the current study provide strong evidence for two discrete *R*. *kanagurta* stocks comprising western Indian Ocean and Southeast Asian populations. The two clades should therefore be considered different management units. While the patterns are clear, results need to be validated because they are based on evidence from a single maternal lineage gene that may not necessarily reflect the complete story.

## Conclusions

The sampled *R*. *kanagurta* populations in Malaysian waters showed only weak spatial differentiation with high genetic variation as inferred from mtDNA Cyt *b*. The study did reveal however, two discrete populations of *R*. *kanagurta*, the Southeast Asian populations (South China Sea, Strait of Malacca, Sulu Sea, Celebes Sea, Andaman Sea) and an Iranian population (western Indian Ocean). In general, however, the data here suggest that *R*. *kanagurta* populations in the IMA and adjacent waters can be regarded as a single stock unit for management purposes. Their inferred demographic history also suggests that *R*. *kanagurta* populations potentially expanded in the late Pleistocene and that gene flow appears to be ongoing among extant populations today.

## Supporting Information

S1 Table Distribution of regionally shared haplotypes for 23 populations of *R*. *kanagurta*. (DOCX)
