# Supplementary material for: High Connectivity in Rastrelliger kanagurta: Influence of Historical Signatures and Migratory Behaviour Inferred from mtDNA Cytochrome b
Source: PLoS One. 2015 Mar 18;10(3):e0119749. doi: 10.1371/journal.pone.0119749 (PMC4365000; doi:10.1371/journal.pone.0119749)
Supplement: S1 Table — (DOCX) [file pone.0119749.s001.docx]

**S1 Table. Distribution of regionally shared haplotypes for 23 populations of *R. kanagurta.***

| Region | Strait of Malacca | | | | | South China Sea 1 | | | | | South China Sea 2 | | | | | | Sulu Sea | | Celebes Sea | | East Indian Ocean | | West Indian Ocean | Total | Haplotype frequency(%)/Total |
| --- | --- | --- | --- | --- | --- | --- | --- | --- | --- | --- | --- | --- | --- | --- | --- | --- | --- | --- | --- | --- | --- | --- | --- | --- | --- |
| Population | KP | TD | TB | BP | KS | TL | PK | C | EN | TS | B | SR | M | BT | KK | VN | KD | SS | SP | ST | A | TH | BA |  |  |
|  |  |  |  |  |  |  |  |  |  |  |  |  |  |  |  |  |  |  |  |  |  |  |  |  |  |
| Hap1 | 1 | 1 |  |  |  | 1 | 1 | 1 | 1 |  |  | 2 |  | 1 | 1 |  |  | 2 | 1 | 2 |  |  |  | 15 | 4.39 |
| Hap2 | 1 |  | 1 | 1 |  |  |  |  | 1 |  |  |  |  |  |  |  |  |  |  |  |  |  |  | 4 | 1.17 |
| Hap5 | 2 |  |  |  |  | 1 |  |  | 1 |  |  |  |  |  |  |  | 1 |  |  |  | 1 |  |  | 6 | 1.75 |
| Hap8 | 1 | 3 |  |  |  |  | 1 |  |  |  | 2 |  | 1 | 1 | 2 | 2 | 3 |  |  |  |  |  |  | 16 | 4.68 |
| Hap12 | 1 |  |  | 1 |  |  |  |  |  |  |  |  |  |  |  |  |  |  |  | 1 | 1 | 1 |  | 5 | 1.46 |
| Hap21 |  | 2 |  |  | 1 |  | 1 | 1 | 2 | 1 |  |  | 2 |  | 1 |  | 1 |  |  |  |  |  |  | 12 | 3.51 |
| Hap28 |  | 1 |  |  |  |  |  |  |  |  |  |  |  |  | 1 |  |  |  |  | 1 |  |  |  | 3 | 0.88 |
| Hap29 |  | 1 |  | 1 |  |  |  | 2 | 2 | 2 | 2 | 1 | 1 |  |  | 1 | 2 |  |  |  |  |  |  | 15 | 4.39 |
| Hap31 |  |  | 1 |  |  | 1 |  |  |  |  |  |  |  |  |  |  |  |  |  |  |  |  |  | 2 | 0.58 |
| Hap32 |  |  | 1 |  |  |  |  | 1 |  |  |  |  | 1 |  |  |  |  |  |  |  |  |  |  | 3 | 0.88 |
| Hap51 |  |  |  |  | 1 | 1 |  |  |  |  | 1 |  |  |  |  |  |  |  |  |  |  |  |  | 3 | 0.88 |
| Hap52 |  |  |  |  | 1 |  |  | 1 | 1 |  |  |  |  |  |  | 1 |  |  |  |  | 1 |  |  | 5 | 1.46 |
| Hap61 |  |  |  |  | 1 |  |  |  |  |  |  |  |  |  |  |  |  |  |  |  |  | 1 |  | 2 | 0.58 |
| Hap69 |  |  |  |  |  | 1 |  | 1 |  |  |  |  | 2 |  |  |  |  |  |  |  |  |  |  | 4 | 1.17 |
| Hap94 |  |  |  |  |  |  |  |  | 1 |  | 1 |  |  |  |  | 1 |  |  |  |  |  |  |  | 3 | 0.88 |
| Hap105 |  |  |  |  |  |  |  |  |  | 1 |  |  |  |  |  |  |  |  |  |  | 1 |  |  | 2 | 0.58 |
| Hap113 |  |  |  |  |  |  |  |  |  |  | 1 | 2 |  | 1 |  |  |  |  |  |  |  |  |  | 4 | 1.17 |
| Hap121 |  |  |  |  |  |  |  |  |  |  |  | 1 |  |  |  |  |  |  |  | 1 |  |  |  | 2 | 0.58 |
| Hap130 |  |  |  |  |  |  |  |  |  |  |  |  | 1 |  |  |  |  | 1 |  |  |  |  |  | 2 | 0.58 |
| Hap146 |  |  |  |  |  |  |  |  |  |  |  |  |  | 1 |  |  |  |  |  |  | 1 |  |  | 2 | 0.58 |
| Total* | 16 | 19 | 12 | 15 | 14 | 15 | 12 | 15 | 15 | 19 | 15 | 13 | 15 | 17 | 16 | 9 | 21 | 15 | 19 | 15 | 13 | 7 | 17 | 342 |  |
| TV:TS | 1:7 | 1:8 | 1:9 | 1:9 | 1:14 | 1:6 | 1:9 | 1:13 | 1:15 | 1:10 | 1:19 | 1:25 | 1:05 | 1:10 | 1:5 | 1:10 | 1:18 | 1:13 | 1:7 | 1:8 | 1:8 | 1:10 | 1:8 |  |  |

Footnotes: Total* - Actual total including singletons and regional specific haplotypes which are not represented in the table.
